# Clinical features and outcomes of foot keloids treated using complete surgical excision and full thickness skin grafting followed by corticosteroid injections

**DOI:** 10.1186/1757-1146-6-26

**Published:** 2013-07-15

**Authors:** Tae Hwan Park, Ji Hae Park, Choong Hyun Chang

**Affiliations:** 1 Department of Plastic and Reconstructive Surgery, Kangbuk Samsung Hospital, Sungkyunkwan University School of Medicine, Seoul, Republic of Korea; 2 Deokjeok Health Care Center, Incheon, Republic of Korea

**Keywords:** Keloid, Foot, Steroid, Recurrence

## Abstract

**Background:**

Keloids are often resistant to treatment and have high recurrence rates. To the best of the authors’ knowledge, however, there have been very few case reports related to foot keloids. The purpose of this retrospective case-series was to summarize the baseline characteristics of a cohort of patients, introduce our treatment regimen for the successful treatment of foot keloids.

**Methods:**

Patients were treated with surgical excision followed by full thickness skin grafting combined with postoperative steroid injections combined with silicone gel sheeting over a period of eight years from December 2004 to November 2012 at our institution. Subjective outcome was evaluated using *Patient Scar Assessment Scales*. The final objective outcome was judged by two independent physicians at the time of 12 months after treatment as recurrence or non-recurrence.

**Results:**

Of 79 patients, 75 (94.9%) were women and 4 (5.1%) were men. The average age was 18 (range 7-43) years. The average pretreatment total size of the lesions was 50 (range 18-150) cm. The number of patients treated for a primary foot keloid was 29 (36.7%), and 70 patients (63.3%) were treated for a recurrent keloid that failed to respond to prior treatments. Prior treatments included single therapies such as surgical excision alone (4 patients, 5.1%), prior steroid injection alone (33 patients, 41.8%), and laser therapy (2 patients, 2.5%). Other therapies included combination treatments (11 patients, 13.9%). Most patients reported improved Patient Scar Assessment Scale by lapse of time. All patients completed the treatment regimen and follow-up of 12 months. Of these patients, 62 patients (78.5%) achieved successful treatment, while the remaining 17 (21.5%) experienced recurrence.

**Conclusions:**

We successfully treated foot keloids using complete surgical excision and full thickness skin grafting followed by four corticosteroid injections (at one month intervals).

## Introduction

**Figure 1:**
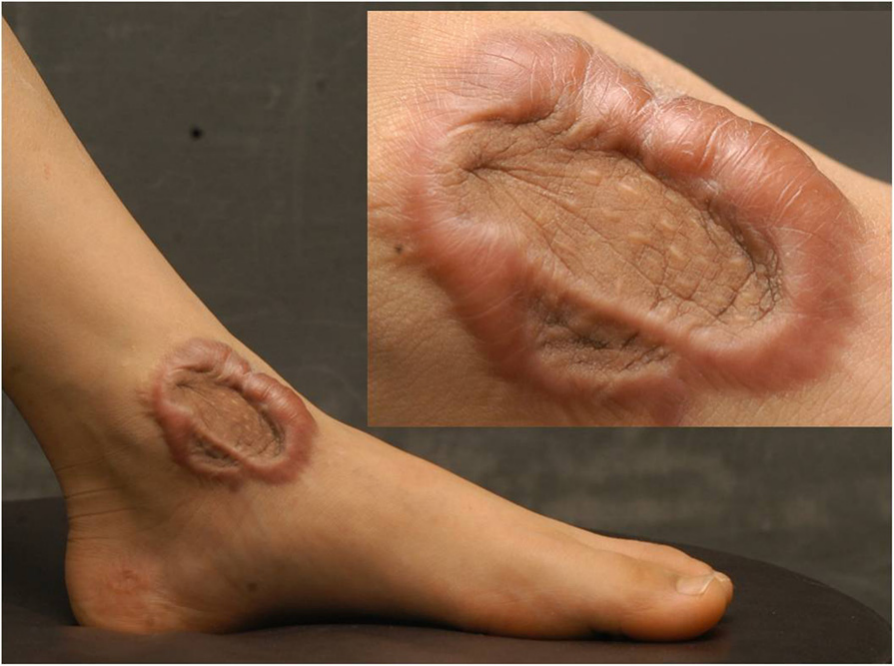
Central sparing in a foot keloid.

**Figure 2:**
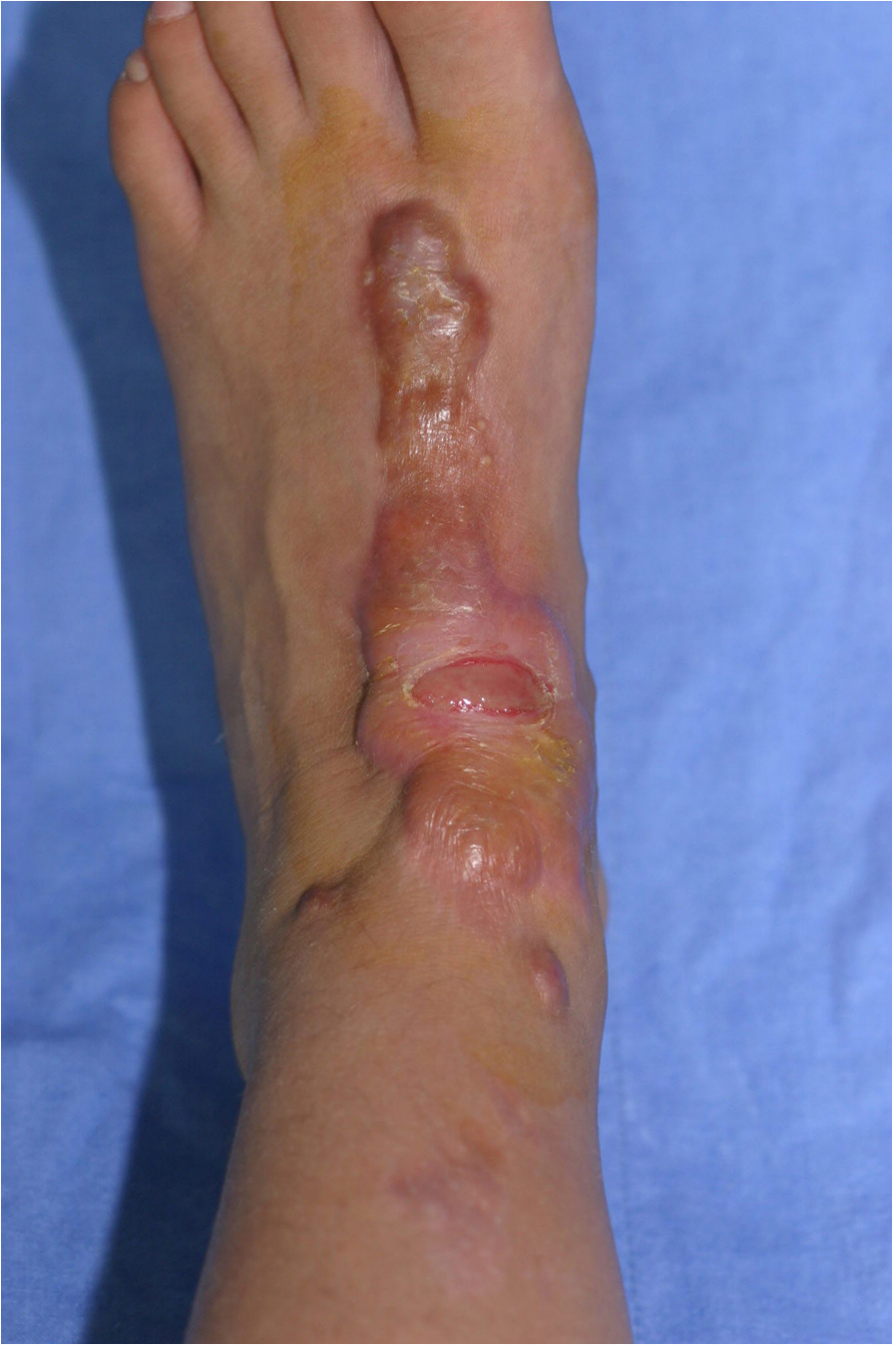
Typical ulceration in a foot keloid.

The proliferation of normal tissue healing processes results in keloid scarring that enlarges well beyond the original wound margins [1]. Keloids are often resistant to treatment and have high recurrence rates. Numerous treatment methods, including surgical excision, cryotherapy, pressure therapy, intralesional corticosteroid injection, radiation therapy, topical silicone-gel sheeting, and laser treatment, have been adopted for the treatment of keloids, suggesting that no single method has surfaced as the accepted standard. Previously, our group has reported extensive experiences of various locations of keloids [2-6]. Morbidity associated with involvement of these anatomical locations typically includes pruritus, pain, tenderness, and cosmetic disfigurement. In the present retrospective study, we report on our experiences with foot keloids. Some of the common characteristics of foot keloids are central sparing (Figure 1) and ulceration (Figure 2). They also typically cause secondary infections, contracture, and limited range of motion, which leads to severe functional problems. To the best of the authors’ knowledge, however, there have been few case reports studying this condition [7-11].

The purpose of this study was to summarize the baseline characteristics of a cohort of patients, introduce our treatment regimen for the successful treatment of foot keloids.

## Methods

### Inclusion and exclusion criteria and study design

Patients were treated with surgical excision followed by full thickness skin grafting combined with postoperative steroid injections combined with silicone gel sheeting over a period of eight years from December 2004 to November 2012 at Kangbuk Samsung Hospital, Seoul, Korea. Patients with foot keloids admitted to our institution for surgical therapy were included in the present study based on several criteria: 1) the presence of clinically definite keloid scars on the foot dorsum; 2) scheduled for a complete surgical excision with full thickness skin grafting; 3) the patient can understand and comply with adjuvant corticosteroid injection therapy. Patients were excluded from the study if they were unavailable for follow-up or if histological confirmation was not obtained. All patients consented to a final follow-up visit 12 months after treatment. We analyzed data including patient age, total size, gender, etiology, previous treatment history and modality, recurrence, and clinical photographs.

### Surgical technique and postoperative care

**Figure 3:**
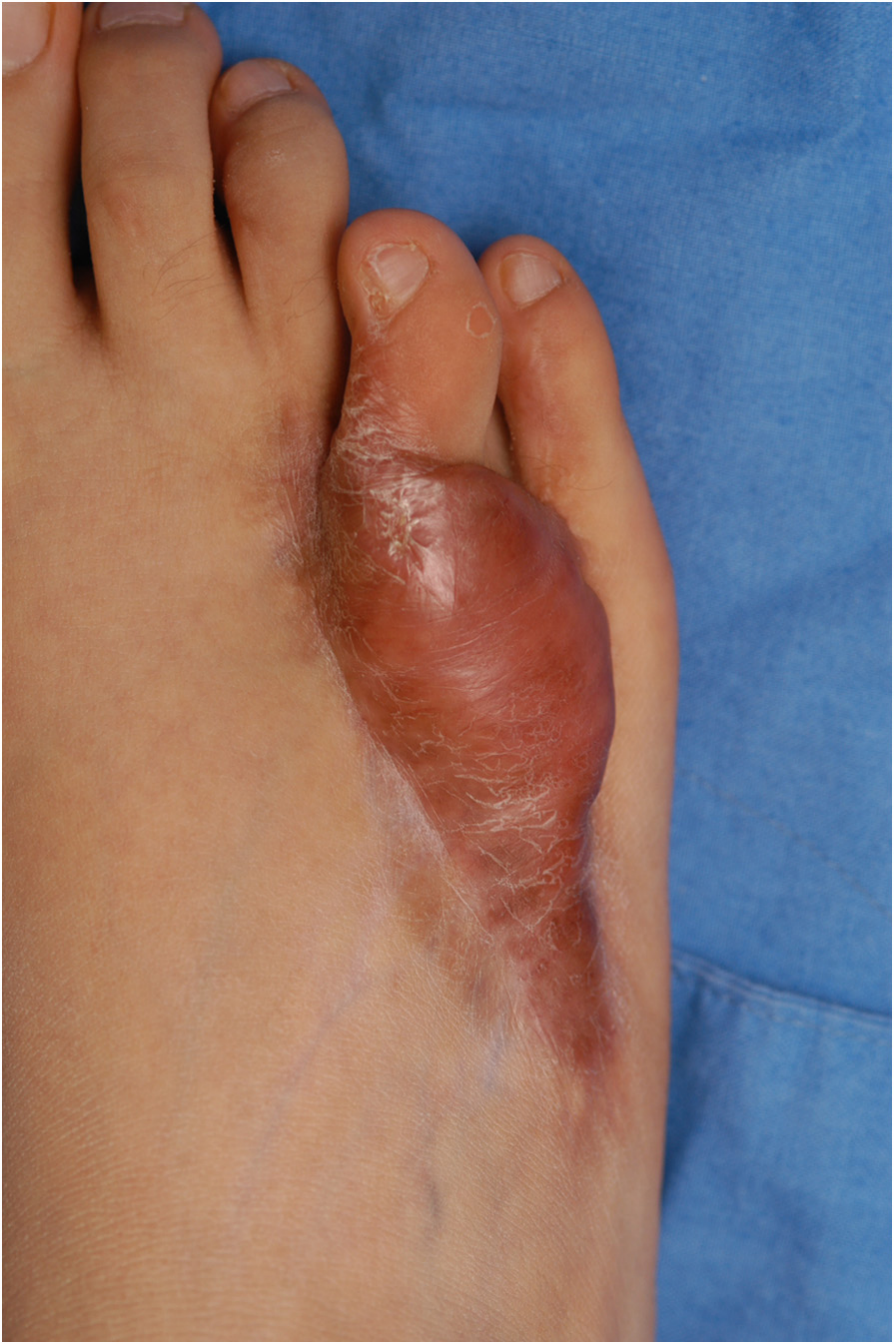
Preoperative appearance of a patient.

**Figure 4:**
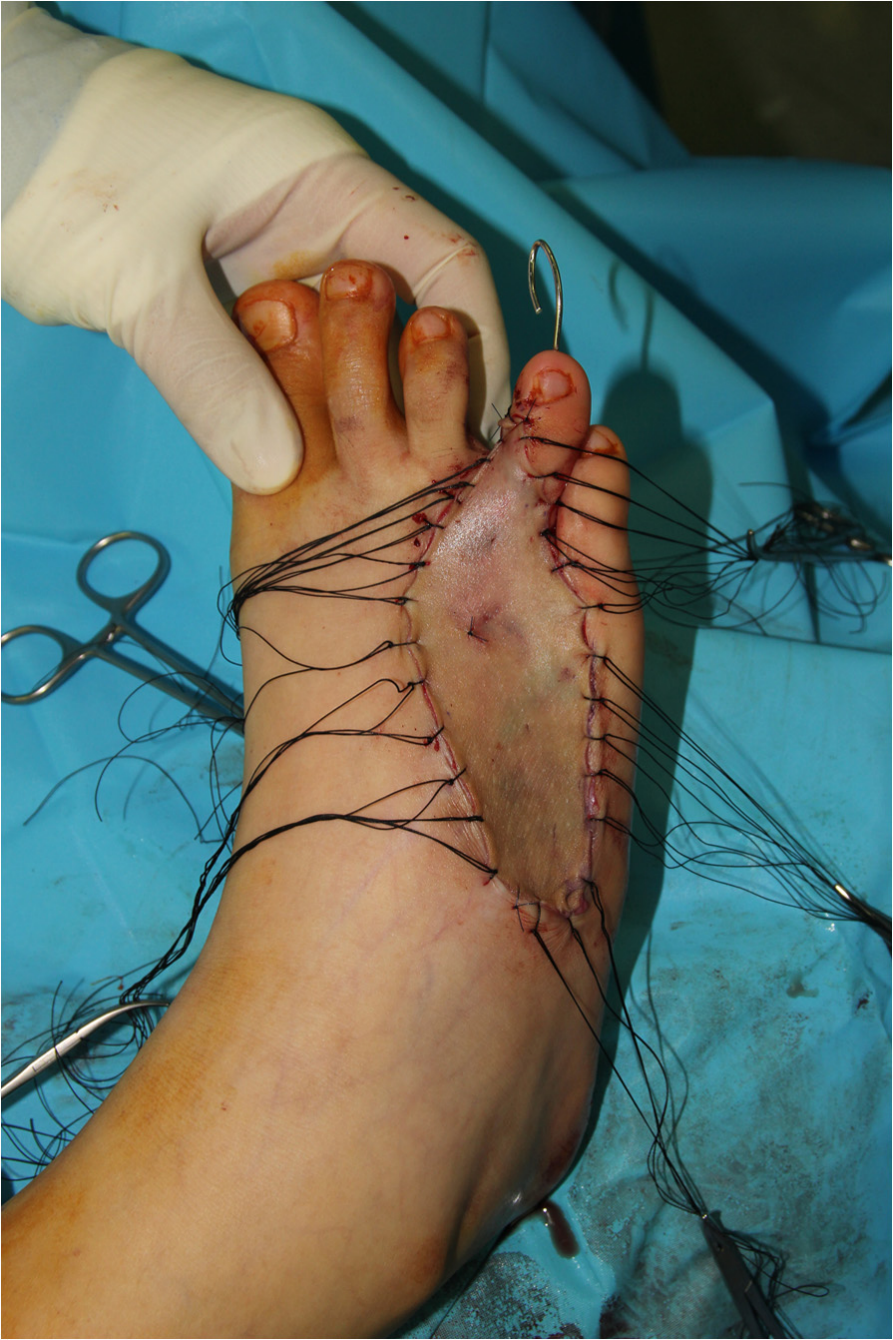
Approximation of the harvested full thickness skin to the defect area using silk 2-0 sutures.

**Figure 5:**
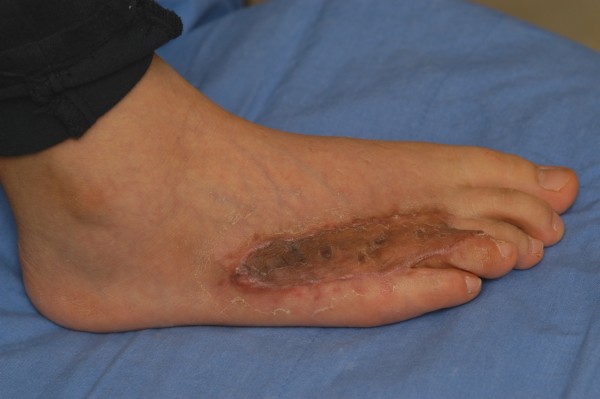
Postoperative 2 months.

**Figure 6:**
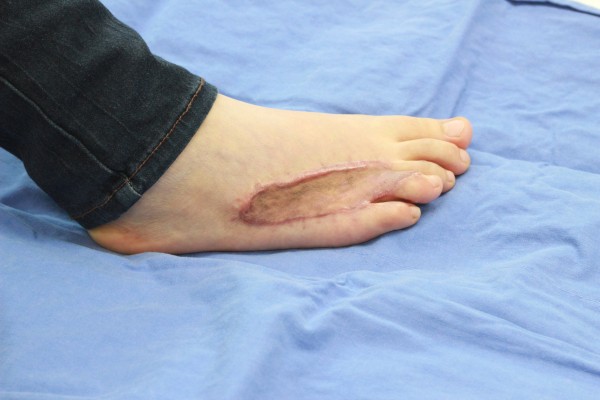
Postoperative 12 months.

All surgical procedures were performed under general anesthesia. We excised foot keloids (Figure 3) with 2-3 mm surgical free margins until we noticed moderate dermal bleeding from the surrounding normal tissue. Bleeding was controlled with bipolar coagulation. We then measured the defect size, closed the defect with full thickness skin grafting harvested from the inguinal area. Skin grafts can generally be designed in an elliptical shape, and when harvesting, we tried not to injure the underlying superficial fatty layer. After trimming the fat tissue tangentially to the skin, we approximated the harvested skin to the defect area using silk 2-0 sutures. (Figure 4) We applied a bolster compressive dressing to optimize the revascularization process for approximately 6-7 days. All keloids were sent for histological examination to confirm the clinical diagnosis. The dressings were taken off for the first time 6-7 days after grafting. All patients were seen 10-14 days postoperatively to remove stitches and again approximately 1, 2, and 3 months postoperatively for corticosteroid injections (Figures 5 and 6).

### Follow-up and outcome assessment

The follow-up period ranged from 12 months to 17 months. During the follow-up period, outpatient consultations were scheduled every one month for the first 3 months, at bimonthly intervals for the next 4 months, and every 4 months for the remainder of the follow-up period until treatment completion. During the first 3 months postoperatively, we adopted the *Patient Scar Assessment Scale* from the *“Patient and Observer Scar Assessment Scales”* (POSAS). An assessment of keloids was initially performed at the time of total stitch removal as baseline, and then again at 1, 2, and 3 months postoperatively. During each visit, treated wounds were clinically assessed using the *Patient Scar Assessment Scale*[12]. The patient scored the characteristics of scar color, pliability, thickness, relief, itching, and pain. All items of the scale were scored numerically on a scale of 1 to 10 with a score of 10 corresponding to the worst possible scar characteristics. The final treatment objective outcome was judged by two independent physicians at the time of 12 months after treatment as recurrence or non-recurrence, with non-recurrence defined as a scar without signs of significant elevation or extension, although slight marginal elevation or redness could be present.

### Statistical analysis

All statistical analyses were conducted using PASW version 18.0 (IBM, Armonk, New York, USA). Our data were not normally distributed, and consequently non-parametric tests were used. Descriptive statistics are presented as medians with ranges or as numbers and percentages. A General linear Model was used to analyze the results of the *Patient Scar Assessment Scale*. Two-tailed hypothesis tests were considered statistically significant if p < 0.05.

## Results

Baseline patient characteristics are presented in Table 1. Of 79 patients, 75 (94.9%) were women and 4 (5.1%) were men. The average age was 18 (range 7-43) years. The average pretreatment total size of the lesions was 50 (range 18-150) cm. The number of patients treated for a primary foot keloid was 29 (36.7%), and 70 patients (63.3%) were treated for a recurrent keloid that failed to respond to prior treatments. These prior treatments included single therapies such as surgical excision alone (4 patients, 5.1%), prior steroid injection alone (33 patients, 41.8%), and laser therapy (2 patients, 2.5%). Other therapies also included combination treatments (11 patients, 13.9%). All patients completed the treatment protocol and the follow-up period of 12 months. Of these patients, 62 patients (78.5%) achieved successful treatment, while the remaining 17 (21.5%) experienced recurrence. The results of the *Patient Scar Assessment Scale* are summarized in Table 2.

**Table 1:** Baseline patient characteristics

	**Total patients (n=79)**
Age, years	18.00 (7 - 43)
Total size, cm^2^	50.00 (18 - 150)
Gender:	
Female, n (%)	75 (94.9%)
Male, n (%)	4 (5.1%)
Previous treatment history:	
No, n (%)	29 (36.7%)
Yes, n (%)	50 (63.3%)
Surgical excision, n (%)	4 (5.1%)
Steroid injection, n (%)	33 (41.8%)
Laser therapy, n (%)	2 (2.5%)
Combination treatments, n (%)	11 (13.9%)
Etiology:	
ORIF d/t fracture, n (%)	54 (68.4%)
1’ closure d/t laceration, n (%)	11 (13.9%)
2’ healing following burn, n (%)	10 (1.1%)
Others, n (%)	4 (5.1%)
Recurrence	
No, n (%)	62 (78.5%)
Yes, n (%)	17 (21.5%)

Values are median(ranges) for continuous variables and number (percentages) for categorical variables.

**Table 2:** Results of patient scar assessment scale

**Patient scar assessment scale**	**Baseline**		**Month 1**		**Month 2**		**Month 3**	
	**Mean**	**SD**	**Mean**	**SD**	**Mean**	**SD**	**Mean**	**SD**
Total score	16.82	4.34	14.09	3.94	13.25	3.89	12.61	3.89
Pain	2.30	1.05	2.15	0.88	2.11	0.85	2.10	0.83
Itchiness	4.09	1.08	3.37	1.04	3.05	0.97	2.92	0.98
Color	3.78	1.53	2.86	1.29	2.66	1.23	2.51	1.18
Stiffness	3.29	1.08	2.72	0.88	2.63	0.87	2.52	0.88
Thickness	3.41	1.04	3.00	1.01	2.78	1.01	2.56	0.98
Irregularity	3.91	1.06	3.32	1.05	3.27	1.03	3.18	0.99

## Discussion

Keloids have a tendency to recur after surgical excision at rates as high as 80-100% [13]. Recently, there have been substantial updates on lower extremity wound coverage. This has been possible due to a higher frequency of trauma in the lower extremity than any other areas in the body. As trauma is one of the main factors for keloid formation, the frequency of keloids in the lower extremity is expected to be high. During the past eight years, we have treated lower extremity keloids based on our strict treatment protocol; complete surgical excision followed by full thickness skin grafting and corticosteroid injections combined with silicone gel sheeting. In fact, lower extremity keloids are rarely reported in the medical literature. Thus, we present 79 foot keloid patients with various causal mechanisms who have been treated at our institution in the past eight years.

Various authors reported the onset of KD to be between the ages of 10 and 30 years, and the incidence is not frequent in the first decade of life [14]. Our present work, however, revealed that foot keloids can frequently happen around the age of 10 years and consequently, the average age of foot keloids (18 yrs, range from 7 to 43 yrs) are lower than those of other keloids. (Average age of chest keloids is 32 yrs [5], ear keloids 24 yrs [4], and face:34 yrs [6] ) This is true if considering the high incidence of lower extremity trauma in elderly patients. Although one can think that most lower extremity keloids can be covered by socks or footwear, most foot keloid patients have significant functional problems such as limited range of motion of the ankle or toes and difficulty in wearing normal footwear due to hypertrophied wounds. For these reasons, the surgeon’s fear of recurrence inspite of treatment or young age of the patient cannot be an appropriate excuse for delayed interventions. Rather, the goal of treatment should focus on relieving the patient’s discomfort and functional disabilities.

**Figure 7:**
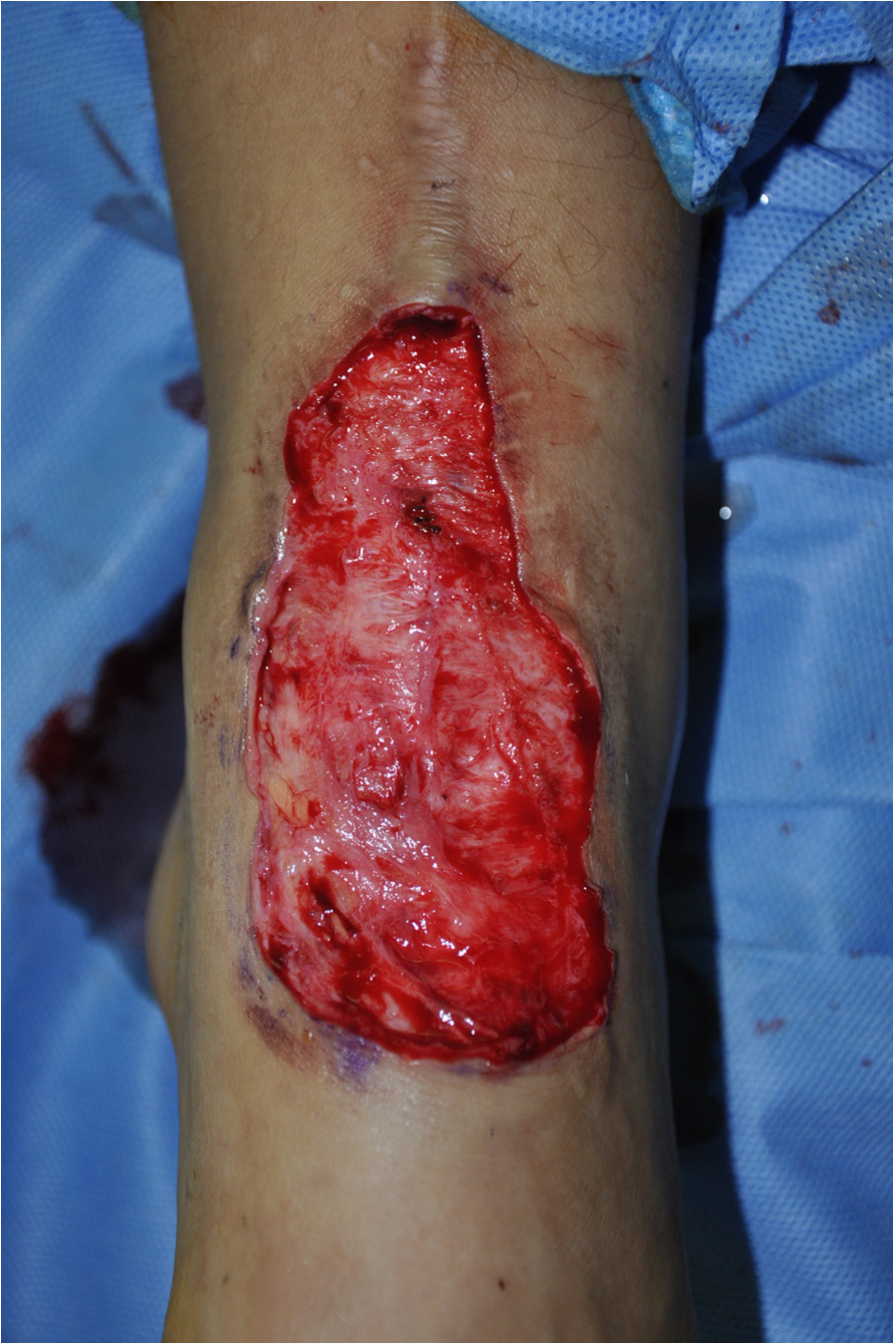
A representative shape of the defect following complete excision that could not be closed primarily.

As defects after excision usually ranges from 3×6 to 10 × 15 cm in size, it cannot be closed primarily (Figure 7). As we usually have 2-3 mm additional surgical free margins, we routinely harvest full thickness skin to completely cover the defect. Spilt thickness skin grafting can increase secondary contracture, while using flaps and neglecting the lower step on the reconstructive ladder can expose additional donor site morbidity due to the high recurrence rate of keloids.

Muneuchi studied the long-term outcome of injection of triamcinolone acetonide into keloid scars in an Asian population [15]. Between 1985 and 2003, they treated 94 patients by injecting 1 to 10 mg of triamcinolone acetonide depending on the size of the lesion at one month intervals [15]. Thirty-one patients (33%) gave up treatment because of pain and the lack of immediate improvement. Although repeated corticosteroid injections are somewhat painful and may cause several complications, such as temporary tissue atrophy, telangiectasia, and hypopigmentation, corticosteroid injection as an adjuvant therapy has low morbidity, is cost-effective, is easy to administer, and provides durable and reliable results. Therefore, we adopted postoperative corticosteroid injection therapy similar to the one described in our previous study [6]. In our earlier experience, we did not use steroid injections in a routine manner. However, we noted increased recurrences at postoperative 3-4 months. Now, we use steroid injections on every foot keloid patient. All patients were seen at 1, 2, and 3 months postoperatively for corticosteroid injections.According to the literature, radiation therapy is an effective adjuvant modality for the treatment of keloids [16,17]. However, we chose to exclude radiation as it is a significant risk factor for non-healing lower extremity wounds. Moreover, as we covered the defect with skin grafting, we were aware that radiation therapy as an adjuvant therapy could cause secondary ulceration or infections. In addition, the average age of lower extremity keloid patients (18 yrs) are relatively young compared to other anatomical areas, and many of these young patients and their parents fear the risk of radiation-induced malignancy [18,19].

After repeated corticosteroid injection therapy, we applied silicone gel sheeting to the wound. The exact mechanism is still unknown, but it is suggested that hydration, occlusion, and increasing temperature affect the collagenase kinetics. Since our patients are mostly older than preschool-age, the application of a silicone gel sheet to the lower extremity wound was not bothersome to the patients [2].

Although keloid recurrence is considered extremely high, only a few studies have focused on the risk factors for keloid recurrence. Multivariate logistic regression analysis of auricular keloids in our previous study revealed that the presence of previous treatment history, high patient body mass index, and low keloid growth rate owing to the longer duration of the disease are clinical risk factors for keloid recurrence [4]. According to the present study, we did not find any significant risk factors for foot keloid recurrence after multivariate logistic regression analysis. (data not shown) According to our clinical experiences, however, a relatively high recurrence was seen on the distal portion of the foot (regions of toes), and on the proximal portion (ankle). On the other hand, the mid portion of the foot is relatively free of recurrence. However, this is not statistically significant.

**Figure 8:**
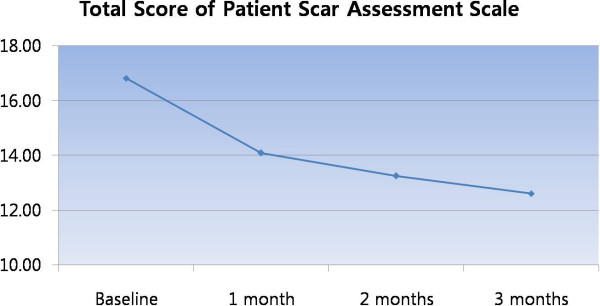
Improved “patient scar assessment scale” by lapse of time.

**Figure 9:**
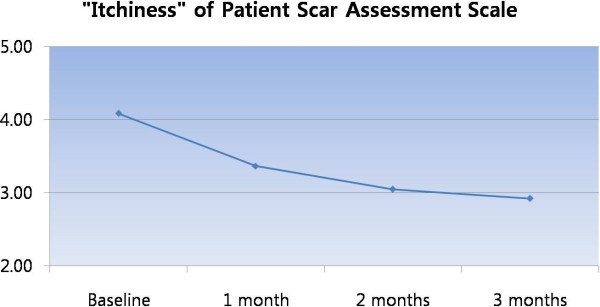
Improved “itchiness” by lapse of time.

Our protocol resulted in a recurrence free rate of 78.5%. Although this result seems relatively low compared to our previous outcome study of other anatomical locations, most patients reported improved Patient Scar Assessment Scale (pain, itchiness, color, stiffness, thickness, and irregularity) (Figure 8). In particular, most patients reported improved itchiness as time progressed up to 3 months postoperatively (Figure 9). Therefore, we can conclude that complete surgical excision with full thickness skin grafting followed by postoperative injection therapy yields excellent outcome. Keloid formation was not seen on the donor site in our clinical practice, which was made possible through strict dermal closure.

This study also had some limitations. First, our patients were all of Korean origin. For this reason, our results cannot be applied to Africans or African Americans. Second, as this study has the characteristics of case-series, the results could be at risk of confounding effects. Accordingly, the effectiveness of the intervention is likely to be over-estimated. Randomized controlled trials or series of single case experimental designs would be warranted to truly test the effectiveness of our treatment protocol.

## Conclusions

Our results demonstrate that we successfully treated foot keloids over a period of eight years using our technique of complete surgical excision and full thickness skin grafting followed by four corticosteroid injections (one month intervals). Scores obtained from the Patient Scar Assessment Scale (pain, itchiness, color, stiffness, thickness, and irregularity) showed trends towards improvement in most participants with particular improvements demonstrated for itchiness.

## Ethical approval

All study protocols used in this study were approved by the Institutional Ethical Committee of Kangbuk Samsung hospital. Our present study is in compliance with the Helsinki Declaration.

## Competing interests

None of the authors has a financial interest in any of the products, devices, or drugs mentioned in this manuscript.

## Authors’ contributions

THP coordinated the study, interpreted the data, contributed to discussion and wrote the manuscript. JHP interpreted and analyzed the data, reviewed, edited and wrote the manuscript. CHC is the guarantor of this work and, as such, had full access to all the data in the study and takes responsibility for the integrity of the data and the accuracy of the data analysis. All authors read and approved the final manuscript.
